# Mathematical Modelling of the Transmission Dynamics of Contagious Bovine Pleuropneumonia Reveals Minimal Target Profiles for Improved Vaccines and Diagnostic Assays

**DOI:** 10.1371/journal.pone.0116730

**Published:** 2015-02-10

**Authors:** Amos Ssematimba, Joerg Jores, Jeffrey C. Mariner

**Affiliations:** 1 International Livestock Research Institute, Old Naivasha Road, P.O. Box 30709, 00100, Nairobi, Kenya; 2 Institute of Veterinary Bacteriology, University of Bern, Bern, Switzerland; 3 Tufts Cummings School of Veterinary Medicine, 200 Westboro Rd. North Grafton, MA, United States of America 01536; Miami University, UNITED STATES

## Abstract

Contagious bovine pleuropneumonia (CBPP) is a cattle disease that has hampered the development of the livestock sector in sub-Saharan Africa. Currently, vaccination with a live vaccine strain is its recommended control measure although unofficial antimicrobial use is widely practiced. Here, modelling techniques are used to assess the potential impact of early elimination of infected cattle via accurate diagnosis on CBPP dynamics. A herd-level stochastic epidemiological model explicitly incorporating test sensitivity and specificity is developed. Interventions by annual vaccination, annual testing and elimination and a combination of both are implemented in a stepwise manner and their effectiveness compared by running 1000 simulations per intervention over ten years. The model predicts that among the simulated interventions, the ones likely to eliminate the disease from an isolated herd all involved annual vaccination of more than 75% of the animals with a vaccine that protects for at least 18 months combined with annual testing (and elimination of positive reactors) of 75% of the animals every six months after vaccination. The highest probability of disease elimination was 97.5% and this could occur within a median of 2.3 years. Generally, our model predicts that regular testing and elimination of positive reactors using improved tests will play a significant role in minimizing CBPP burden especially in the current situation where improved vaccines are yet to be developed.

## Introduction

Contagious bovine pleuropneumonia (CBPP) is a cattle disease caused by *Mycoplasma mycoides* sub. *mycoides* (*Mmm*) and is transmitted through direct and repeated contacts between infected and susceptible animals [1]. Its economic implications are of great significance to both farmers and to national economies in sub-Saharan Africa. Outbreaks in previously disease-free areas together with increased incidences in endemically infected areas have been observed [2] and its epidemiology in these countries is influenced by both social and cultural factors [3–5] which complicates its control.

In a study involving twelve countries in sub-Saharan Africa, Tambi et al. [6] estimated losses due to CBPP as 3.7 million euros per country annually. But this is very likely to be an underestimation since the authors used the prevalence numbers provided by the countries. The latter do not reflect the situation on the ground simply because coverage of screening in most countries is far from optimal due to restricted funds. The disease has continued to impact the livestock industry as evidenced with recent outbreaks in Uganda [7] and Kenya [8] and the reported prevalence trend in Tanzania [9]. Jores et al. [10] showed that CBPP cases had been reported in most sub-Saharan Africa countries in the period between 2010 and 2013.

CBPP screening and diagnosis depends on either conventional isolation of the etiological agent or serological and molecular tests. The complement fixation test (CFT) and a competitive enzyme-linked immunosorbent assay (cELISA) are the two primary serological tests recommended by the world organization for animal health (OIE) [11] and are both characterized by wanting test sensitivity and specificity [12].

Vaccination using attenuated *Mmm* strains such as T_1_44 or T_1_SR is the recommended control measure against CBPP [13]. Yet these vaccines confer only partial and short-lived protection and elicit adverse effects on the host [13]. In addition, there seems to be limited knowledge about the importance of vaccination among the farmers [14]. Given the short duration of vaccine protection, annual booster vaccination would be the solution [15] but this is hampered by logistical and financial limitations. Hence these vaccination campaigns have failed as an increase in both the disease prevalence and distribution has been observed in tropical Africa [10]. Besides, there is limited adoption of quarantine measures because of social, economic and cultural factors thus rendering disease control difficult [3,4]. Consequently, alternative disease management strategies that are pro-rural and pro-poor farmers are proposed and include developing rapid, inexpensive and accurate diagnostic tools, improving field surveillance through regular testing and implementing a “monitored-on-farm slaughter” of infected animals as well as increasing farmer awareness. The choice of alternative strategies to be implemented, either singly or in combination, requires careful assessment of their effectiveness.

Mathematical models provide the means to generate evidence-based information on infectious disease control and play an important role in understanding the dynamics of infectious diseases [16,17]. They can be used to guide predictive and contingency planning during epidemics as well as to guide the assessment of the efficiency and effectiveness of newly proposed control strategies [18]. Currently, development of novel diagnostic point-of-care tests is ongoing and in view of that, this study develops a mathematical model that explicitly incorporates diagnostic test sensitivity and specificity with an underlying aim of assessing the potential impact of improved diagnostic tools on the field surveillance of CBPP and to guide deployment of testing and elimination combined with vaccination in CBPP management.

## Materials and Methods

### Model

**Fig 1:**
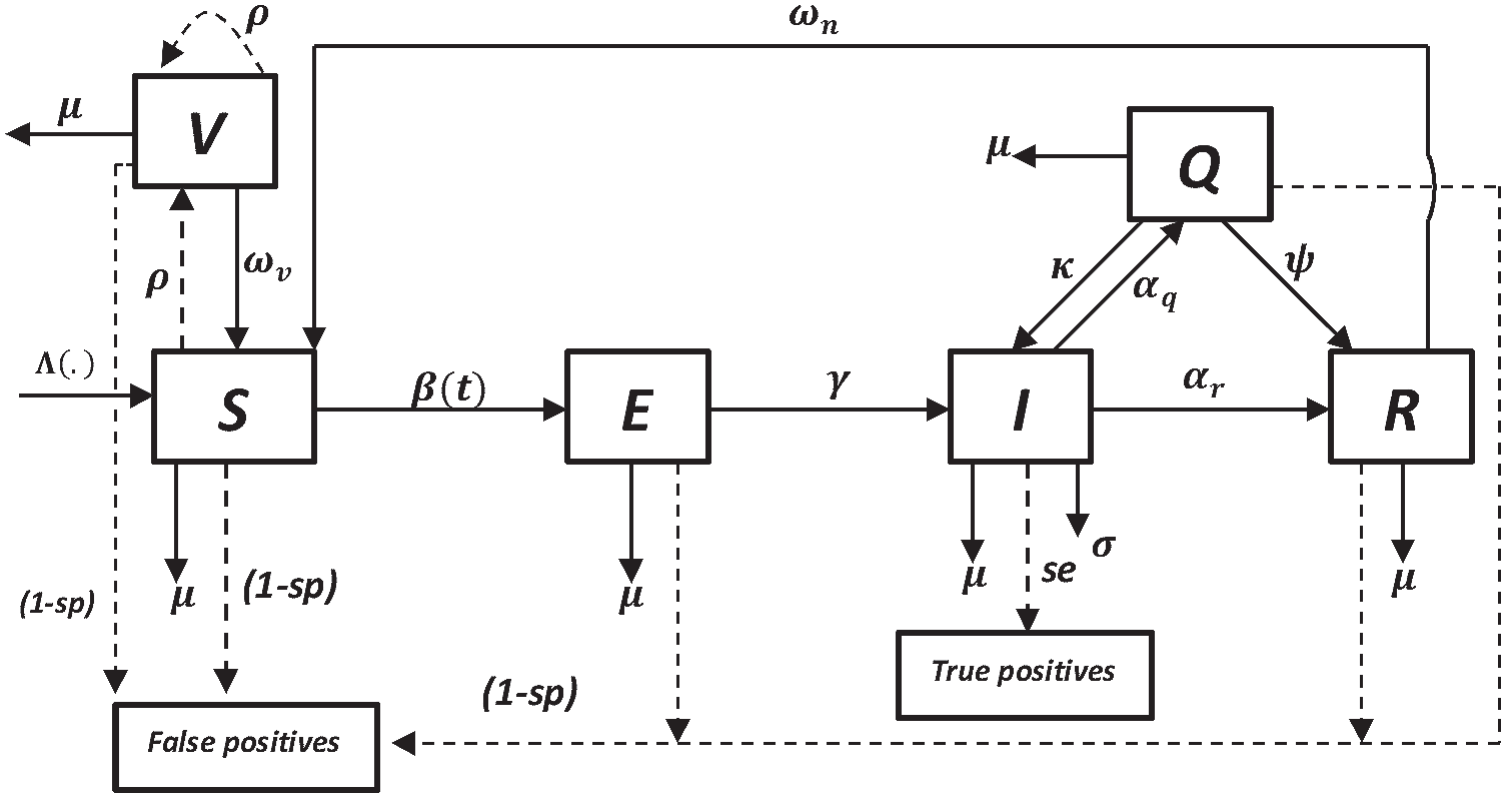
A compartmental model for the dynamics of CBPP that incorporates testing and elimination of positive reactors. The model is of the type “SVEIMQR” where *S* represents the susceptible, *V* the vaccinated, *E* the exposed, *I* the infectious, *M* the eliminated, *Q* the chronically infected and *R* the recovered state. Testing and vaccination are discrete time events and are thus represented by the dashed arrows and all the other events are continuous and are represented by the non-dashed arrows.

We model the dynamics of CBPP within an isolated herd with the individual animals as the epidemiological units of interest. The modelled processes are captured in the compartmental model shown in Fig. 1. The model is an adapted and enhanced version of that developed by Mariner et al. [19,20] and still assumes a seasonal transmission term.

We incorporated annual pulsed vaccination [21] and pulsed testing and elimination of positive reactors. To assess the potential role of improved diagnostic tools, we explicitly incorporated test specificity (sp) and sensitivity (se) into the model as done previously by Barlow et al. [22]. In the model, when implementing the pulsed intervention strategies, all animal-categories were targeted. However, vaccination in all other compartments is ignored since the current vaccine is known to only confer a temporally immunity to the S- animals and may “boost” the titre level in the V- animals (since we assume a gamma distributed waning of vaccine-induced immunity). Additionally, as a consequence of test characteristics–CFT only detects active infection and doesn’t detect antibodies in vaccinated animals one month post vaccination–we assumed that all positive reactors were false positive except those from the I- compartment. In order to maintain a constant herd size, all outflows from the herd were countered by an equivalent inflow, albeit of only susceptible animals.

Briefly, the adjusted model is of the form “SVEIMQR” where *S*(*t*) are susceptibles, *V*(*t*) are vaccinated, *E*(*t*) are latently infected, *I*(*t*) are infectious, *M*(*t*) are eliminated positive reactors, *Q*(*t*) are chronically infected and *R*(*t*) are the recovered animals. The deterministic-equivalent of the system depict in Fig. 1 in which a frequency-dependent transmission is assumed is here presented.

$$\frac{dS}{dt}=\text{Λ}(.)-\frac{\beta (t)SI}{N}-\rho \sum_{n=0}^{\infty }S(nT^{-})\delta (t-nT)-\epsilon (1-sp)\sum_{n=0}^{\infty }S(nT^{-})\delta (t-nT)-\mu S+w_{r}R+w_{v}V$$ (1.1)

$$\frac{dV}{dt}=\rho \sum_{n=0}^{\infty }S(nT^{-})\delta (t-nT)-(w_{v}+\mu )V-\epsilon (1-sp)\sum_{n=0}^{\infty }V(nT^{-})\delta (t-nT)$$ (1.2)

$$\frac{dE}{dt}=\frac{\beta (t)SI}{N}-(\gamma +\mu )E-\epsilon (1-sp)\sum_{n=0}^{\infty }E(nT^{-})\delta (t-nT)$$ (1.3)

$$\frac{dI}{dt}=\gamma E+\kappa Q-[\alpha_{q}+\alpha_{r}+\sigma +\mu ]I-\epsilon se\sum_{n=0}^{\infty }I(nT^{-})\delta (t-nT)$$ (1.4)

$$\frac{dM}{dt}=\epsilon se\sum_{n=0}^{\infty }I(nT^{-})\delta (t-nT)+\epsilon (1-sp)\sum_{all\;X}\left(\sum_{n=0}^{\infty }X(nT^{-})\delta (t-nT)\right)$$ (1.5)

$$\frac{dQ}{dt}=\alpha_{q}I-(\kappa +\psi +\mu )Q-\epsilon (1-sp)\sum_{n=0}^{\infty }Q(nT^{-})\delta (t-nT)$$ (1.6)

$$\frac{dR}{dt}=\psi Q+\alpha_{r}I-(w_{r}+\mu )R-\epsilon (1-sp)\sum_{n=0}^{\infty }R(nT^{-})\delta (t-nT)$$ (1.7)

Where, generally, $Y(nT^{-})=\underset{\xi \to 0}{lim}Y(nT-\xi ),\xi >0$ is the left-hand limit of *Y*(*t*), and *δ*(*t*) is the Dirac delta-function and *X* = {*S*,*V*,*E*,*Q*,*R*}. Vaccination and testing are applied as impulses at discrete times *t* = *nT* (with *n* = 0, 1, 2, …) and the moment immediately before the n-th pulse is denoted as *t* = *nT* ^-^ [21]. The transmission term is of the form *β(t) = β*_0_ (1+ *β*_1_sin(2 πt)) where *β*_0_ represents the average effective contact rate, *β*_1_ is the amplitude of seasonality and *t* is time scaled to days. Recruitment into the herd is adjusted to ensure that all losses due to elimination of positive reactors and due to disease-induced mortality are compensated for and is now given by the term$\Lambda (.)=bN+\frac{\sigma }{365}I+\frac{\epsilon }{365}[se\;I+(1-sp)\sum X]$.

A gamma distribution was used to model the waning of vaccine-induced immunity [23–25]. The distribution had eight subclasses to represent the eight-month duration of vaccine-induced immunity [26]. The model was implemented stochastically in Mathematica 9.0 (Wolfram Research, Inc.) using a Gillespie’s direct algorithm [16].

Most of the disease-specific parameters (their definitions and estimation) were obtained from [19]. These and other parameters are presented in Table 1. In the model simulations, as in [19], all other parameters except the vaccination proportion and the tested fraction were entered as pert probability distributions to cater for the possible uncertainty in their estimates. This distribution is a version of a beta distribution that is parameterized by the minimum, mode and maximum parameter value. The initial herd size was set at 500 heads of which 2% are infectious, 2% are latently infected, 55% are recovered and the rest are susceptible as in Mariner et al. [19]. Animals that recover from CBPP are assumed resistant to further challenge [5,27] and here, as in [19], these animals are assumed to be protected for a period of ten years. The default targeted vaccination proportion is set at 60% in accordance with the reported ranges of 20–60% [28] and 52–77% obtained in a field trial conducted in Kenya [29]. CFT specificity and sensitivity were set to 98% and 64% respectively [30].

**Table 1:** Parameter estimates used in the model simulations (all rates are per day).

Parameter	Minimum	Mode	Maximum
Sequestration rate (*α*_*q*_)	0.011	0.013	0.018
Recovery rate (*α*_*r*_)	0.0036	0.0045	0.0059
Effective contact rate (*β*_0_)	0.07	0.126	0.13
Transition rate from *E* to *I* (*γ*)	0.018	0.024	0.036
Sequestrum reactivation rate (κ)	0.00007	0.00009	0.00011
Natural mortality rate (μ)	0.00050	0.00055	0.00062
Default vaccination coverage (*ρ*)^a^ *	---	0.375	---
CBPP-induced mortality rate (*σ*)	0.0064	0.0090	0.013
CBPP-induced immunity waning rate (*ω*_*n*_) **	---	0.00027	---
Vaccinal immunity waning rate (*ω*_*v*_) *	---	0.0042	---
Sequestrum resolution rate (*ψ*)	0.0068	0.0075	0.0079
Test sensitivity (*se*) *	---	0.64	---
Test specificity (*sp*) *	---	0.98	---
Herd testing coverage (*ϵ*)*	---	0.75	---
Seasonality coefficient (*β*_1_)	---	0.5	---
Recruitment rate (*b*)	0.00050	0.00055	0.00062

*New parameters in the model (also described in the main text); all others are as reported in Mariner et al. [19]

**Waning rate of CBPP-induced immunity is set to be very low to mimic a life-long disease-induced immunity

^a^ Obtained by taking the product of target vaccination proportion and vaccine-protected fraction (efficacy) i.e., 0.6x0.625

For each intervention scenario, 1000 simulations running for ten years were performed. The scenarios were compared based on their effect on: the chance of eliminating CBPP from the herd (where “elimination chance” technically refers to the proportion of simulations in which the disease got eliminated from the herd), the time it took to clear CBPP from the herd and the cumulative number of fatal cases. For testing and elimination interventions, we also looked at the number of false negative animals for the different test sensitivities.

In addition to the above indicators, we also assessed the required vaccination level to eradicate the disease using the theory of basic reproduction number (*R*_0_), where the critical vaccination coverage (*v*_c_) is given by *v*_*c*_ = 1–1/*R*_0_ [16].

### Intervention Scenarios

In a stepwise control measure implementation approach, the intervention-free dynamics of the disease were simulated as the baseline scenario followed by simulations with: a) pulsed annual vaccination alone (AV), b) pulsed annual testing and elimination alone (AT), and c) a combination of pulsed annual vaccination and testing and elimination (AVT). Annual pulsed interventions were incorporated as single time-step events, one day in this case, with one-year long intervals between consecutive exercises. In all simulations where applicable, the first vaccination exercise occurred at the start of the simulation while the first testing exercise occurred after six months. In the text, intervention scenarios are labelled using the format: “Strategy type: _ percentage vaccinated _vaccine protection duration _tested fraction_ test sensitivity. For example, scenario “AVT:vac37.5%_8months_test75%_se64%” represents an annual vaccination and testing strategy in which the effective vaccination coverage is 37.5% with a vaccine that protects for eight months together with testing of 75% of the animals with a 64% sensitive test.

Vaccination proportions of 37.5%, 75% and 90% with the current vaccine that induces an eight months vaccine protection period were investigated. The vaccine-induced protection period was increased to 18 and 24 months in order to determine the quality of vaccine needed to clear CBPP at herd level. Under the annual testing strategy, two testing proportions; = 75% and 95% were compared and test sensitivity was increased to 95% to assess the potential impact of an improved test on the disease dynamics. Lastly, under the annual vaccination and testing strategy, the “current” vaccination coverage of 37.5% (i.e., 62.5% 60% in Table 1) was compared with improved vaccination campaigns where coverage was increased to 75% and 90% with vaccines that protect the animal for 8, 18 and 24 months in combinations with annual testing (and subsequent elimination of positive reactors) of 75% of the animals with two test sensitivities: se = 64% and 95%.

## Results

**Fig 2:**
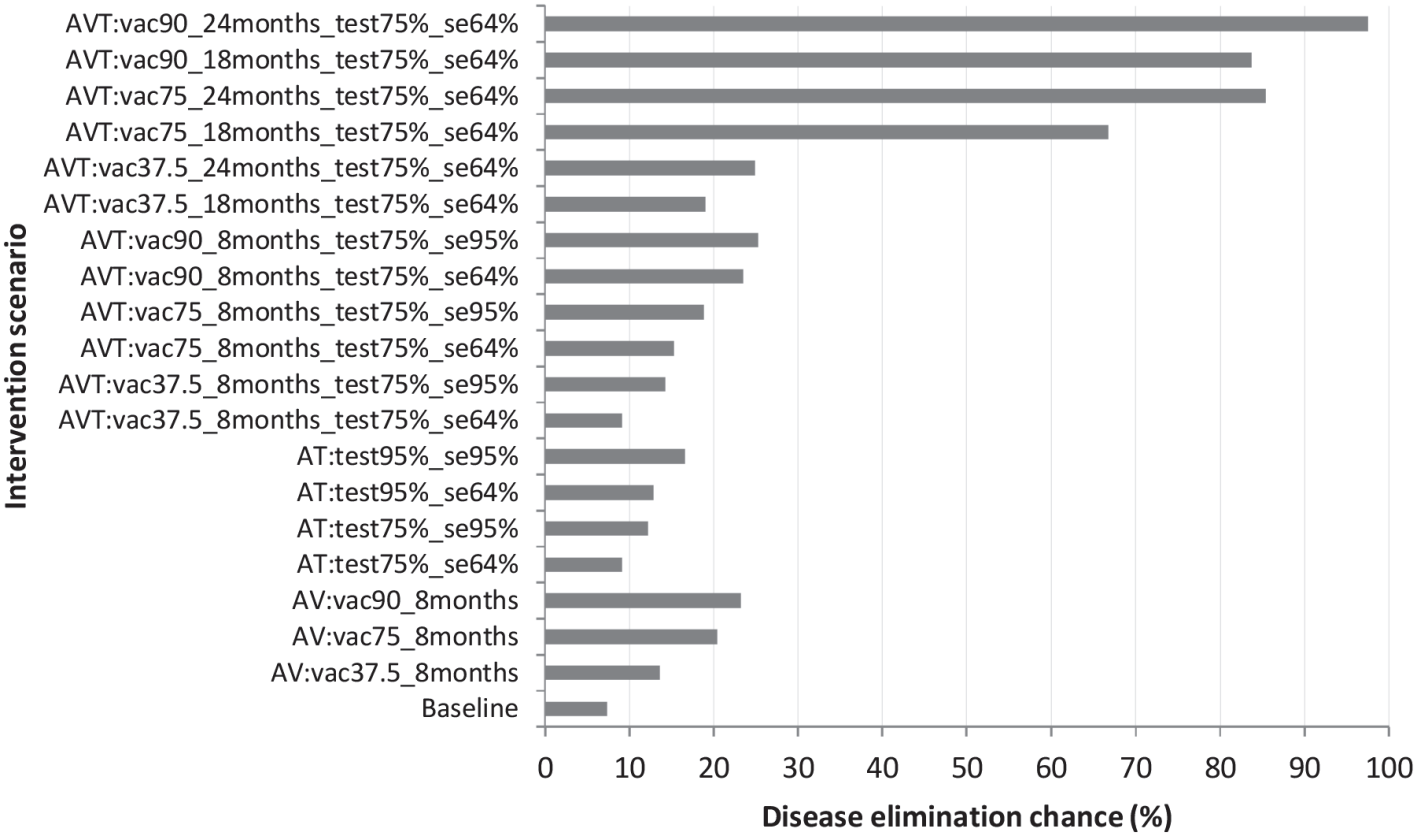
Disease elimination chances for the different intervention scenarios. Elimination chance represents the fraction of simulations in which the disease dies out before the end of simulation time. As an example, scenario “AVT:vac37.5%_8months_test75%_se64%” represents an annual vaccination and testing strategy in which the effective vaccination coverage is 37.5% with a vaccine that protects for eight months together with testing of 75% of the animals with a 64% sensitive test.

**Fig 3:**
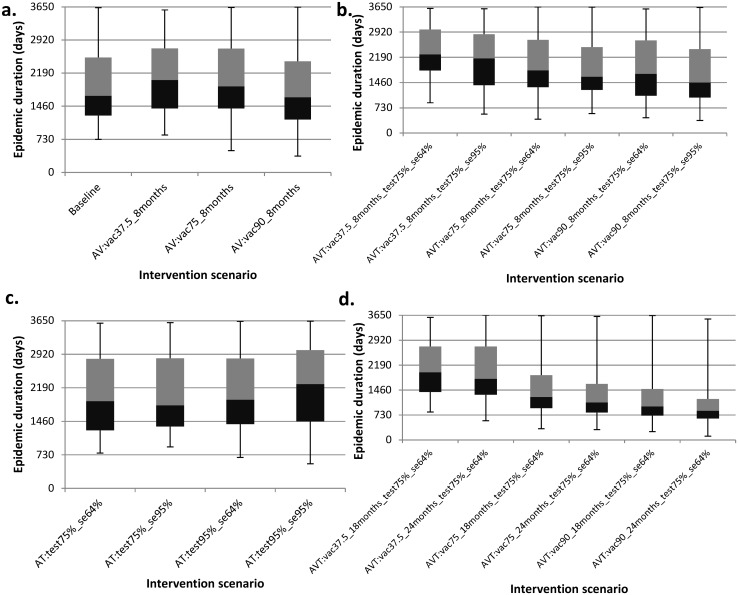
Boxplots for the predicted effects of the various intervention scenarios on the epidemic duration. Panel (a) presents epidemic durations under the baseline and annual vaccination (AV) strategies. Panel (b) depicts epidemic durations under the annual vaccination and testing (AVT) strategy for different test sensitivity and vaccination coverage. Panel (c) presents epidemic durations under the annual testing (AT) strategy for varying test sensitivity and tested fraction. Panel (d) shows epidemic durations under the annual vaccination and testing (AVT) strategy for varying vaccine protection duration and vaccination coverage. As an example, scenario “AVT:vac37.5%_8months_test75%_se64%” represents an annual vaccination and testing strategy in which the effective vaccination coverage is 37.5% with a vaccine that protects for eight months together with testing of 75% of the animals with a 64% sensitive test.

**Fig 4:**
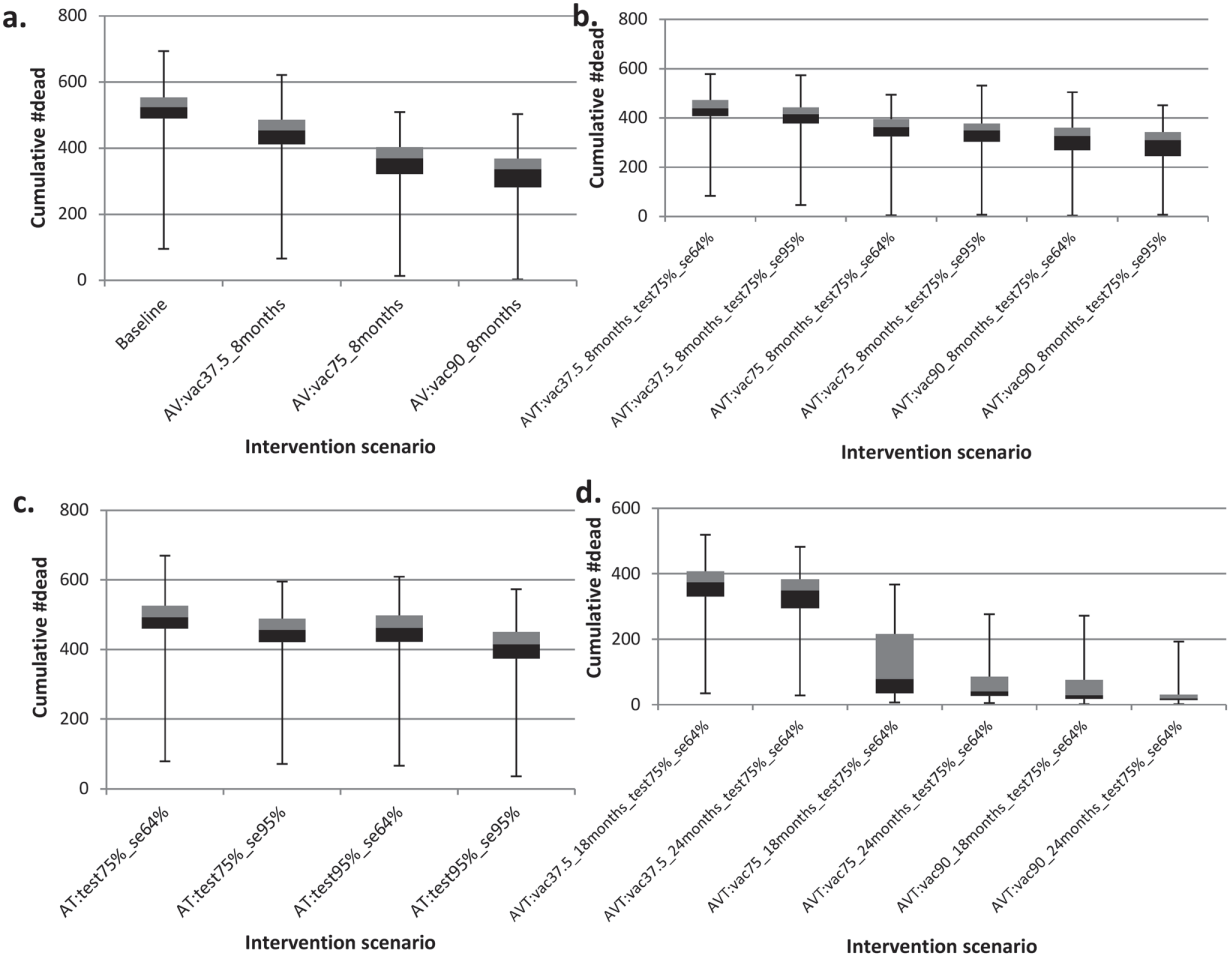
Boxplots for the predicted effects of the various intervention scenarios on the cumulative number of fatalities. Panel (a) presents the cumulative number of fatalities under the baseline and annual vaccination (AV) strategies. Panel (b) depicts the cumulative number of fatalities under the annual vaccination and testing (AVT) strategy for different test sensitivity and vaccination coverage. Panel (c) presents the cumulative number of fatalities under the annual testing (AT) strategy for varying test sensitivity and tested fraction. Panel (d) shows the cumulative number of fatalities under the annual vaccination and testing (AVT) strategy for varying vaccine protection duration and vaccination coverage. As an example, scenario “AVT:vac37.5%_8months_test75%_se64%” represents an annual vaccination and testing strategy in which the effective vaccination coverage is 37.5% with a vaccine that protects for eight months together with testing of 75% of the animals with a 64% sensitive test.

**Fig 5:**
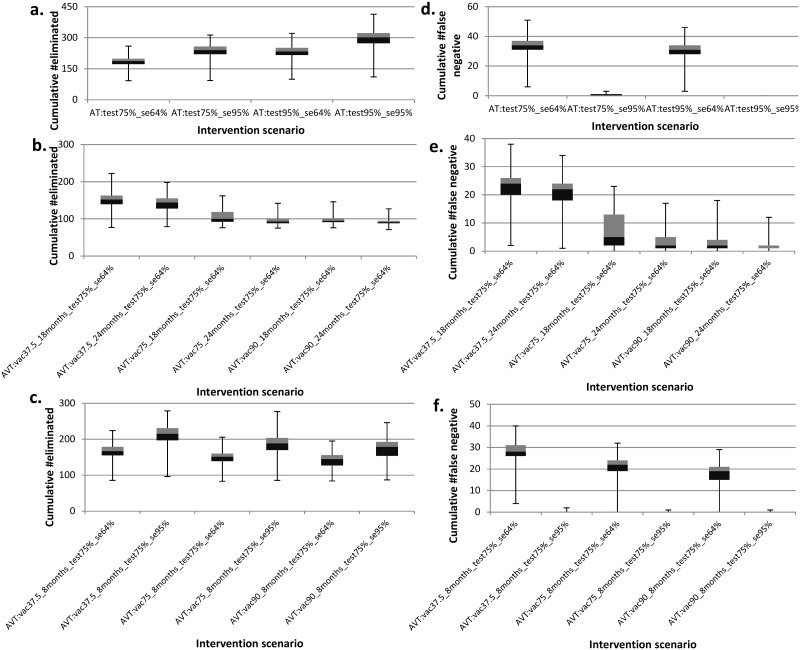
Boxplots for the predicted effects of the various intervention scenarios on the cumulative number eliminated (panel a-c) and false negative animals (panel d-f). Panel (a) presents the cumulative number eliminated under the annual testing (AT) strategy for varying test sensitivity and tested fraction. Panel (b) depicts the cumulative number eliminated under the annual vaccination and testing (AVT) strategy for varying vaccine protection duration and vaccination coverage. Panel (c) presents the cumulative number eliminated under annual vaccination and testing (AVT) strategy for varying test sensitivity and vaccination coverage. Panel (d) presents the cumulative number of false negative animals under the annual testing (AT) strategy for varying test sensitivity and tested fraction. Panel (e) depicts the cumulative number of false negative animals under the annual vaccination and testing (AVT) strategy for varying vaccine protection duration and vaccination coverage. Panel (f) presents the cumulative number of false negative animals under annual vaccination and testing (AVT) strategy for varying test sensitivity and vaccination coverage. As an example, scenario “AVT:vac37.5%_8months_test75%_se64%” represents an annual vaccination and testing strategy in which the effective vaccination coverage is 37.5% with a vaccine that protects for eight months together with testing of 75% of the animals with a 64% sensitive test.

**Fig 6:**
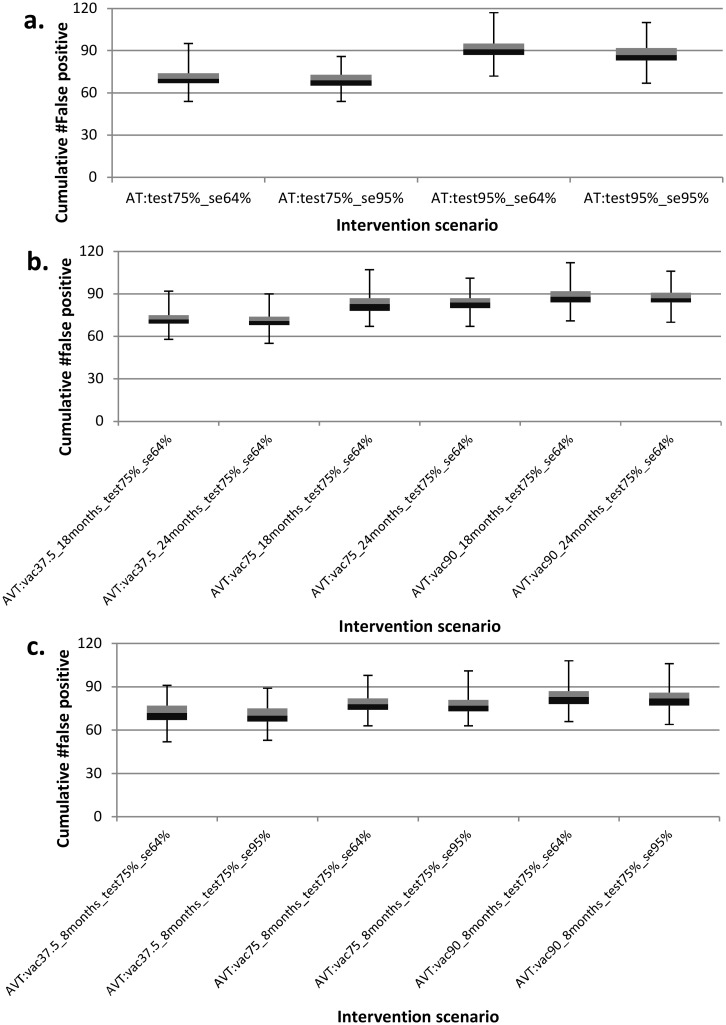
Boxplots for the predicted effects of the various intervention scenarios on the cumulative number of false positive animals. Panel (a) presents the cumulative number of false positive animals under the annual testing (AT) strategy for varying test sensitivity and tested fraction. Panel (b) depicts the cumulative number of false positive animals under the annual vaccination and testing (AVT) strategy for varying vaccine protection duration and vaccination coverage. Panel (c) presents the cumulative number of false positive animals under annual vaccination and testing (AVT) strategy for varying test sensitivity and vaccination coverage.

We present results from the model predictions for an isolated herd (i.e., a herd with no interactions with other herds) using the model parameters displayed in Table 1. The simulated data is presented in S1 Data. Fig. 2 presents the model predictions for the proportion of simulations in which the disease died out before the end the simulation period for the different intervention scenarios simulated. All other results are presented as boxplots depicting the median, 25^th^and 75^th^ percentiles, minimum and maximum predicted values from the 1000 simulations. Fig. 3 depicts epidemic durations and Fig. 4 presents the cumulative number of animals that succumbed to the disease. Fig. 5 shows the cumulative number of animals eliminated and false negative animals and lastly, the cumulative number of false positive animals is presented in Fig. 6.

On the potential effect of (any sort of) intervention, simulating the disease dynamics revealed that with no intervention at all (baseline scenario), there was a 7.4% chance of eliminating CBPP from an isolated herd (Fig. 2) and this could occur within a median of 4.6 years (Fig. 3) with cumulatively 524 fatalities (Fig. 4). This scenario had the highest number of disease-induced deaths and the lowest disease elimination chance compared to all other scenarios. However, its disease elimination time frame was shorter than when intervening with all the simulated annual vaccination strategies with less than 90% efficacy, and was also shorter than all annual testing scenarios as well all combined strategies except those involving greater than 75% vaccination efficacy.

Note that different intervention scenarios affect the performance indicators differently; hence determining the best scenario would require a cost-benefit analysis that is beyond the scope of this study. Following we assess the interventions based on their effect on the various performance indicators selected for this study.

### Effect of interventions on disease elimination chance and time to clear disease

Among the three simulated strategies under the annual vaccination strategy, immunizing 90% of the population at risk with a vaccine that protects for only eight months had the highest chance of eliminating the disease at 23.2% and the least at 13.6% was realised when immunizing 37.5% as currently practiced (Fig. 2). Pertaining the time it takes to clear the disease from the herd, all simulated intervention scenarios under the annual vaccination strategy had the disease maintained in the herd beyond four years (Fig. 3).

Under the annual testing strategy, the lowest elimination chance was 9.1% and it arose from testing of 75% of the animals with the available test that is 64% sensitive and 98% specific while the highest was 16.6% arising when 95% of the animals were tested with an improved test that has 95% sensitivity (Fig. 2). The lowest median time taken to clear the disease from the herd for the simulated annual testing strategies was five years and could be achieved by testing (and eliminating positive reactors) 75% of the animals using an improved test with 95% sensitivity (Fig. 3).

Among the simulated scenarios, the only intervention strategies that could have disease elimination chances that are beyond 25% were among the combined annual vaccination and testing strategies. In relation to disease elimination chance, the top four strategies (among the simulated) all involved annual vaccination of more than 75% of the animals with a vaccine that protects for at least one and half years combined with annual testing of 75% of the animals every six-months after vaccination (test specifications: 64% sensitivity and 98% specificity). The chances ranged from 66.7% to 97.5% (Fig. 2) and their corresponding disease elimination times ranged from 2.3 to 3.5 years (Fig. 3) with elimination time decreasing with increasing chance.

### Effect of interventions on the cumulative number of fatalities

Under the annual vaccination strategy, with reference to the baseline scenario (which resulted in 524 fatalities over ten years), the model predicts that by implementing vaccination using the current live vaccine (that induces good protection for eight months) with the current efficacy of 37.5%, the median cumulative number of fatalities was reduced by 13.4% and increasing the efficacy to 75% and 90% would reduce these fatalities by 29.6% and 35.9% respectively (as computed from median values in Fig. 4).

Among the simulated annual testing strategies, annual testing of 75% of the animals with the current test that is 64% sensitive and 98% specific resulted in the highest cumulative number of fatalities (N = 493) while the lowest fatalities (N = 415) was realised upon improving the test sensitivity to 95% and the tested fraction to 95% (Fig. 4).

Annual vaccination of at least 75% of the animals with a vaccine that protects for at least 18 months combined with annual testing of 75% of the animals every six-months after vaccination (test specifications: 64% sensitivity and 98% specificity) reduced the cumulative fatalities by a range of 84.9% to 96.4% in reference to the baseline (N = 524 fatalities). Notably, annually vaccinating 90% conferring them immunity for two years combined with annual testing (and elimination of positive reactors) of 75% with the current test that is 64% sensitive would result cumulatively in only 19 fatalities (Fig. 4). However, in the same strategy, a lower vaccination level of 37.5% would at best reduce the fatalities by 33.4%.

### Effect of interventions on the cumulative number of eliminated, false negative and false positive animals

Under the annual testing strategy, testing 75% of the animals with the current test (sensitivity = 64%) resulted in the lowest median cumulative number eliminated at N = 187 (Fig. 5) among which N = 70 were false positives (Fig. 6) and it yielded the highest number of false negatives cumulatively at N = 34 (Fig. 5). Increasing both the test sensitivity and the tested fraction to 95% increased the median cumulative number eliminated to N = 302 with 87 false positives and this strategy yields no false negatives.

For the combined strategies, all strategies involving an improved test (i.e., with sensitivity = 95% and specificity = 98%) yielded no false negatives. With the current test, it is only the scenarios involving annual vaccination of at least 75% of the animals with a vaccine that protects for at least 18 months combined with annual testing of 75% of the animals that could have a maximum of five false negatives but this number was also as low as one.

### On the required critical vaccination coverage for herd-immunity

Concerning the required vaccination coverage to achieve herd-immunity, by using *R*_0_ = 4.5 [19] we estimated the critical vaccination coverage (*v*_*c*_) for disease elimination in a herd as 77.8%. This implies that the protected fraction in the herd should be maintained above that value between successive vaccination rounds. Yet, in-between vaccination events, the vaccine-protected fraction may fall below the required (critical) vaccination coverage thereby ensuring persistence of infection.

## Discussion

Given the current CBPP situation is sub-Saharan Africa, control measures as currently practiced including vaccination did not prevent spread of the disease in recent years. There is need to develop integrated intervention packages to improve CBPP control and to achieve progressive control on the African continent. In this study, mathematical modelling techniques were used to set and assess performance parameters for improved diagnostics assays, vaccines and vaccination regimes that may result in better control of the disease.

While CBPP prevalence was decreasing during the joint vaccination campaign of CBPP and rinderpest [5,31], our model predicts that the chances of eliminating CBPP at a herd level using the currently available vaccines given the current vaccination coverage are minimal.

Our model predicts that annual vaccination as currently practiced in most sub-Saharan Africa countries results only in herd-level advantage in terms of minimizing production losses (reduced morbidity and mortality). Besides having a very low chance of eliminating the disease from the herd at 13.6%, it maintains the disease longer in the herd (compared to not intervening at all). This finding is in line with that of Lu et al. [32] on the potential impact of an imperfect vaccine. Waning of immunity is practically a source of new susceptibles. This replenishment directly impacts on the disease duration in the herd because the disease can only be maintained in herd if there is a sufficient number of susceptibles.

Our model predicts that, we can only have more than 50% chance of eliminating CBPP at a herd level through a combined strategy and with a relatively high annual vaccination of more than 75% of the animals at risk with a vaccine that protects for more than 18 months together with testing and elimination of positive reactors, targeting at least 75% if the currently available test (sensitivity = 64% and specificity = 98%). This coverage is high and can only be attained through improved supervision and incentivisation of vaccination campaigns and efficiency both of which are likely to be better achieved through private and elective vaccination [19,20]. Note however that privatization may increase vaccine utilization over the present situation, but may require additional farmer persuasion [14]. Vaccination coverages beyond 75% can be achieved relatively easily in developed countries [33] but may be unrealistic for most of the sub-Saharan Africa countries due to poor infrastructure in the relatively hard-to-reach farming areas as well as the pastoral and semi-pastoral farming systems. In such conditions, vaccine costs are less than their delivery costs by far, thus longer-protecting vaccines will provide a more realistic option in that region.

Furthermore, our model predictions emphasize the need for a combined strategy if CBPP is to be controlled. The best performing intervention scenario among those simulated involves regular testing and elimination of positive reactors and hence better diagnostic assays are necessary. The model predicts that a test with improved sensitivity, in this case around 95%, is required. As revealed by the simulations, these improved assays will eliminate false negatives that would otherwise go unnoticed and consequently prolong the disease in the herd. Ideally, point-of-care tests that can be used in the field without special equipment and trained staff would increase the proportion of animals to be tested. Additionally, our model predictions emphasize the need to develop a vaccine that protects significantly longer than the current live vaccine. Striving for a higher coverage of vaccination combined with testing is a goal that can be addressed right now while the development of a better vaccine requires researchers to develop and implement an improved vaccine, which may take several years [10].

We note that the predicted case-fatality rate in all simulated scenarios was around 30% and was invariant since all the simulated interventions implicitly had the same effect on both the cumulative number of new cases and the corresponding fatalities. Notably, this rate is higher than the range of 10% estimated in endemic zones [5], for example the 16.5% estimated in a field study conducted in Ethiopia [34]. We hypothesize that, in the field, this value may be, among others, influenced by the undocumented interventions such as early slaughter and antimicrobial use.

On the predicted relatively low chances of CBPP elimination from an isolated herd (i.e. a herd with no interaction with other herds and all recruits being assumed susceptible) with majority of the simulated (Fig. 2), we argue that the observed CBPP persistence at a herd level is on one hand a consequence of disease re-introduction and repeated interaction between herds and on the other hand a consequence of continued influx of (susceptible) animals in a bid to maintain the herd size.

On the possibility of recruiting already (latently) infected animals, we recommend that, whenever there are new recruits into the herd, they should be screened first and all negative reactors be first kept in isolation until such a time (preferably equal to CBPP latent period) that the farmer is sure that the animals are truly CBPP-free and all positive reactors should be rejected. But, on the other hand, the recruited non-infected animals play a significant role in maintaining the transmission chain and it would be worthwhile to postpone these replenishments to until when the herd is deemed free from disease.

Clearly, vaccination as currently practiced in most sub-Saharan Africa countries is below the required critical vaccination coverage of 77.8% that is estimated from the disease’s basic reproduction number. In order to maintain that vaccination level, longer-protecting vaccines are needed and/or the interval between successive vaccination campaigns be reduced. The former requires more input in terms of technology development while the latter is likely to be constrained by logistics.

Note that in order to clearly depict the differences in efficacies between the various intervention scenarios without masking them in the model complexity, between-herd interactions were ignored here. However, in endemic situations, these interactions may be inevitable [5] and with them in place, herd level, let alone national level, CBPP elimination can only be realized if interventions are synchronized at a regional level. This necessitates governments’ intervention through their respective state veterinary services, most of which are dilapidated and underfunded, thereby posing a big challenge to this effort.

All in all, our model predicts that optimal herd-level CBPP elimination can only be achieved through a combined strategy with a clear indication that the development and use of improved vaccines and diagnostic assays is inevitable. It is important to note however that in this study, intervention scenarios were only assessed based on their effect on the disease dynamics aspect and not based on their cost-effectiveness. We therefore recommend that a cost-benefit analysis of those interventions be carried out to scientifically guide the selection of which ones to be adopted in the policy guidelines on CBPP management from the economics perspective. Note also that we assumed that all positive reactors were immediately eliminated from the herd; presumably through slaughter. Since elimination of positive reactors may be non-feasible to implement in pastoral and semi-pastoral farming systems, it is worthwhile to investigate other interventions such as their treatment and reintegration into the herd. In view of this, we recommend that an in-depth study on the potential role of antimicrobial treatment of the positive reactors on the disease dynamics and its economic consequences be carried out.

## Supporting Information

S1 Data The data that is generated from the simulations from which Figs. 2–6 are produced. (XLS)
